# The reliance on conceptual frameworks in qualitative research – a way forward

**DOI:** 10.1186/s12874-025-02461-0

**Published:** 2025-02-13

**Authors:** Joanna E. M. Sale, Leslie Carlin

**Affiliations:** 1 Musculoskeletal Health and Outcomes Research, Li Ka Shing Knowledge Institute, St. Michael’s Hospital, Unity Health Toronto, 30 Bond Street, Toronto, ON M5B 1W8 Canada; 2 Institute of Health Policy, Management & Evaluation, University of Toronto, Health Sciences Building, 155 College Street, Suite 425, Toronto, ON M5T 3M6 Canada; 3 Department of Surgery, Temerty Faculty of Medicine, University of Toronto, 149 College Street, 5th Floor, Toronto, ON M5T 1P5 Canada; 4 Department of Physical Therapy, Temerty Faculty of Medicine, University of Toronto, 160-500 University Avenue, Toronto, ON M5G 1V7 Canada

**Keywords:** Qualitative research, Role of theory, Conceptual frameworks, Data analysis, Critical appraisal, Reflexivity

## Abstract

**Background:**

While acknowledging that theory can be critical to scientific progress, we are concerned about instances of its tendency to encroach on, or replace, deep engagement with data in qualitative research. We discuss conceptual frameworks’ role in conducting and teaching qualitative research.

**Methods:**

We address three questions about our attachment as researchers to theory through conceptual frameworks: (1) What do conceptual frameworks offer qualitative research?; (2) Why do researchers use and teach conceptual frameworks in qualitative research?; and (3) How can we practice and teach rigour while integrating conceptual frameworks in qualitative research?

**Results:**

One way that theory may be misused in qualitative research is in the development and reliance on conceptual frameworks as a prescription for data collection and analysis. We suggest possible ways forward to ensure rigour while integrating frameworks in qualitative research, such as examining the evolution of our own theoretical perspectives.

**Conclusions:**

We need to impart to our students the value of thinking deeply about their own data, of knowing what came before, and of taking the time and making an effort to unite these strands into novel and interesting results.

## Introduction

In 2005, Oxman and colleagues proposed “The OFF theory of research utilization” in the Journal of Clinical Epidemiology [1]. In this witty, well-argued, and entertaining commentary, the authors claim that in the realm of quality improvement and continuing education, we are “drowning in theories” and need less theorizing and more simple logic and common sense. Our purpose now, nearly two decades later, is to revisit this commentary and apply it to our experiences in qualitative health research and teaching qualitative health research methodology. We observe that far from a reduction in theorizing, the past 17 years have seen instead both an increase in its deployment and, we argue, a thinning of its meaning. In particular, in medical and applied health sciences research, the idea of theory has led qualitative researchers and teachers of qualitative research to rely on theory packaged as conceptual frameworks.

While theory informs and advances science in the form of a framework, it may encroach on, or even replace, deep engagement with data in qualitative research and become a prescription for data collection and analysis. We propose potential reasons for this trend and consider other ways to conduct, teach, and supervise qualitative research in medicine and the health sciences.

## Conceptual frameworks in qualitative research

The role of theory in qualitative research has been documented extensively. Our intent is not to describe or summarize this literature, but to critically examine the ways in which conceptual frameworks are used and taught, based on our experiences with conducting research in qualitative health research and with teaching qualitative methodology.

Theory “provides the categories through which we think about and experience the world” [2]. When we theorize, we are “working from within categories of existing thought to radicalize those categories, reveal[ing] their internal contradictions and shortcomings, and demonstrat[ing] their unrecognized possibilities” (p. 252–253). We can therefore understand theory as a reconsideration of the taken-for-granted character of the social world and constituting how we access the world [2].

In this paper, we ask, and attempt to answer, three questions about our attachment as researchers to theory in the form of conceptual frameworks: (1) What do conceptual frameworks offer qualitative research?; (2) Why do researchers use and teach conceptual frameworks in qualitative research?; and (3) How can we practice and teach rigour while integrating conceptual frameworks in qualitative research?

## What do conceptual frameworks offer qualitative research?

Conceptual frameworks represent global ideas about phenomena; they are systematic structures that are made up of concepts and propositions that provide organization for thinking, observing, and interpreting what is seen [3]. These frameworks focus on the concepts in use and are often depicted in diagrammatic representation [4]. A conceptual framework forces the researcher to be selective by deciding which concepts are most important, which relationships are likely to be most meaningful, and what information should be collected and analyzed [5]. Hudon [6] summarized conceptual frameworks as overarching representations of key elements, and the interrelationships among these elements, to use and acknowledge in the process of designing and implementing knowledge translation interventions. Graham and colleagues refer to conceptual frameworks as an “action plan for moving research findings into practice” [3]. Conceptual frameworks can affect every aspect of the study, including the research design [7], determining how to frame the purpose and problem, data collection, and how to make sense of the data [8]. Discussion of, and reliance on, conceptual frameworks appears to have increased in the early 2000s, in conjunction with the growing field of implementation science. In 2005, Eccles and colleagues [9] proposed that the science of implementation research should be improved significantly by a systematic approach to the use of theory so that we could understand why interventions work or do not work. This paper focuses more broadly on the use of qualitative research and conceptual frameworks in health-related fields.

## Why do researchers use and teach conceptual frameworks in qualitative research?

We propose three main explanations for the appeal of using and teaching conceptual frameworks in qualitative research. First, frameworks encourage identifying specific components, sequential stages, and contextual factors to facilitate the implementation of interventions [6]. Frameworks also support the efforts to evaluate the impacts of such interventions. They provide shared terminology [6]. In short, they aim to create order and structure.

The second reason that frameworks appeal in qualitative research is that the process of deduction is likely more seductive than that of induction. According to Miles and Huberman [5], highly inductive studies make good sense when experienced researchers have plenty of time, or are dealing with complex social phenomena. However, these authors suggest that individuals new to qualitative research may wish to pre-structure their methods (with something like a framework) because it reduces the amount of data and simplifies the required analytic work.

Finally, frameworks may be appealing because they allow and even encourage researchers to slot their data into contained categories, which in turn allows and encourages metrics creation. An example is interrater reliability [10], a measure sometimes used to demonstrate methodological rigour and imply higher quality. In so doing, qualitative research submits itself to judgment on criteria that mimic those used in quantitative approaches.

We suggest, however, that these three apparently laudable reasons for relying on conceptual frameworks also have drawbacks. They promote efficiency and comparability, but risk losing what is valuable in good qualitative research: depth. They constitute a false economy.

The first reason, that such frameworks efficiently create order and structure, is a reasonable one – yet, simultaneously, could lead to misguided conclusions. Qualitative data are inherently messy. To derive sense and meaning from them, researchers need to embrace the messiness and the disorder, to become deeply familiar with their data, whether they consist of interviews, observations or text, or a combination of these sources. A pre-selected framework that guides understanding of the data from the outset is tempting to teach and employ, but will often miss or ignore what does not fit.

Similarly, the second proposed benefit that frameworks fit well with deductive reasoning and thus simplify the analytic work is an appealing prospect. We are not arguing that induction and deduction are problematic. We believe that qualitative research has scientific status because it is both inductive and deductive [11]. However, we do want to suggest careful consideration of what is lost during the process of simplification with the deductive use of frameworks. Induction relies not on a pre-selected conceptual framework but on accumulating knowledge and experience against which to lay new information. Trainees in the social sciences of health acquire the ability to analyze data using primarily an inductive approach through well-supervised trial and error and reading. The suggestion by Miles and Huberman that trainees “pre-structure” their data collection – i.e. be guided by a selected framework – to reduce their dataset and thus simplify analysis may lead to speedier outcomes, but not robust ones. Epidemiologists teaching qualitative methods do not suggest that students unfamiliar with statistics leave relevant numerical data out of their analysis to speed it up. Creativity and the ability to develop novel interpretations during analysis is also lost with the urge to pre-structure one’s data. As Bird [12] reminds us, deductive arguments proceed in a way that their assumptions or premises entail their conclusions.

Finally, the argument that the application of conceptual frameworks across datasets, researchers, and areas of inquiry allows comparability in the form of inter-rater reliability and other metrics may result from insecurity on the part of qualitative researchers. Qualitative methods have merit and rigour of their own and rarely benefit from mimicking or echoing those of quantitative research. Transposing concepts such as inter-rater reliability into qualitative investigation does little to ensure or improve the data analysis’ value, richness, or meaning. As Morse [10] advises, researchers need to trust themselves as well as each other in this regard. “[I]t is death to one’s study to simplify one’s insights, coding and analyses so that another person may place the same piece of datum in the same category” (p. 447). Experienced qualitative researchers acknowledge and embrace uncertainty, holding it critical to understanding social phenomena and human actors; we manage uncertainty through reflexivity, dialogue, and transparency. However, these are not quick processes either to learn or conduct. Thus, delivering excellent training in qualitative research methodology is critical.

## How can we practice and teach rigour while integrating conceptual frameworks in qualitative research?

How can we practice and teach rigourous collection and analysis of qualitative data in medicine and the health sciences when we come from different disciplines and may have very different goals in terms of research output? According to Nilsen [13], frameworks are like checklists and serve a more descriptive purpose by highlighting factors believed or found to influence outcomes. Such an understanding of the purpose of investigation diminishes the ability of qualitative researchers to elevate analysis to open-minded interpretation and thus, does a disservice to qualitative research.

Timmermans and Tavory suggest that qualitative researchers should strive to cultivate surprise in their work, rather than be satisfied with simplicity [14]. This process is a combination of chasing “the usual suspects”, which requires knowledge of the theoretical landscape, while remaining open to the “new and unexpected” [14].

Conceptual frameworks can limit the potential of the researcher to remain open to the new and unexpected and tend to confine data into pre-determined categories and relationships. We caution against their overuse and inappropriate application in qualitative investigation. To be useful, it is imperative that students understand that data may extend beyond and not fit the chosen framework. Thus, while conceptual frameworks can be used to inform data collection, we propose that there should be some evidence that data collection extends beyond the frameworks. Topic areas of the interview or observation guide, and questions and/or observations within these topic areas, should not be constrained by framework domains. The aim is for researchers to minimize what Kvale refers to as a “manipulative dialogue” where the interview guide is an instrument for providing the researcher with descriptions and narratives which are then reported according to the researcher’s interest [15]. Coding may reflect the question the researcher chose to ask but also must proceed inductively. We suggest that the coding template include codes that are developed inductively and not only codes that adhere to the framework domains or elements. Interpretation of the data should not be stifled by the conceptual framework; the framework should not monopolize the interpretation and lead the interviewer to frame what a participant says in the researcher’s own theoretical schemes (15). One option may be that researchers report separately the data collected and analyzed in relation to the framework from the data collected and analyzed using a more inductive orientation. Finally, we propose that the study discussion return to the conceptual framework(s). For example, the discussion might note the usefulness of the framework in interpreting the data but also the challenges in relying on the framework for analysis.

We consider other possible ways to move forward. We propose that qualitative researchers stand their ground when it comes to adopting conceptual frameworks, and develop and maintain confidence in their ability to understand their data and work through appropriate analysis. Theory should contextualize but not drive conclusions. Some qualitative research projects, such as projects about quality improvement, program evaluation or policy, may benefit more from conceptual frameworks, while others will gain more from openness and discovery.

On a final note, the frames that we give our research problems shape what we look for and what we see, “as well as what we do not look for or see...[and the]  standpoints from which we start shape what we see and what we view as truth” [16](p. 983-4). While Sandelowski describes using key concepts of frameworks to serve as organizing data analysis, such frameworks may also prematurely close off recognition of other ways to organize the data [17]. In our opinion, and as implied previously, frameworks are suitable for determining potential interview guide topic areas and questions, and perhaps, categorizing or organizing some of the data collected. Knowledge of frameworks can be critical in prompting discussions about what has been previously reported as important in the topic area. While frameworks can serve as a reference for discussions about what was unexpected in the research results, they may not be particularly helpful in interpreting one’s data [18]. This pitfall is especially risky for students and trainees. At some point, these novice researchers will be required to move the analysis beyond the theoretical frame [18].

What happens when we step away from the reliance on frameworks and unbind the trusses linking data to theory in the form of frameworks? We are forced to trust in the expertise of the researchers and provide excellent training to our students and to our rising new researchers in order to ensure they have knowledge of their field. It is not a result that can be achieved by taking short-cuts. Qualitative researchers in the health field need to take a deep dive into disciplinary roots that provide them with the foundations to understand data in the context of what has come before, and rely less on frameworks that increasingly resemble instruction manuals. Even as mature practitioners of qualitative research, we find it essential to continually examine our own theoretical perspectives, their genesis and evolution, to question the assumptions they entail, and, if necessary, to adjust our perspective and practice. The ‘decolonization’ movement in anthropology is a powerful example of such (overdue) self-monitoring. These steps, we suggest, will enhance the practice and the teaching of rigourous qualitative research.

## Conclusion

When a conceptual framework drives a qualitative research project, gaining that understanding may come at a high cost. Charmaz’s [19] sensitizing concepts are broad theoretical orientations, such as symbolic interactionism, that give researchers starting points for developing, rather than limiting, analytic ideas. Her proposal that qualitative research offers an opportunity for transformation [16] suggests to us that this opportunity is not mapped in advance by a framework. Instead, we should allow the research journey to transform us [16]. We need to impart to our students the value of thinking deeply about their own data, of knowing what came before, and of taking the time and making the effort to unite these strands into something novel and interesting. The process will be slower and messier than it would be while relying on a particular framework, and the result may not turn the world on its head, but it will increase our knowledge of it.

## Data Availability

Not applicable.
